# Neurological and vestibular findings in three cases of Multiple Sclerosis

**DOI:** 10.1590/2317-1782/20232021153en

**Published:** 2023-11-20

**Authors:** Alanna Stefany de Lima Evangelista, José Diniz, Ana Paula Machado Costa, Mário Emílio Teixeira Dourado, Erika Barioni Mantello

**Affiliations:** 1 Programa Associado de Pós-graduação em Fonoaudiologia, Universidade Federal do Rio Grande do Norte – UFRN – Natal (RN), Brasil.; 2 Departamento de Cirurgia, Universidade Federal do Rio Grande do Norte – UFRN – Natal (RN), Brasil.; 3 Hospital Universitário Onofre Lopes, Universidade Federal do Rio Grande do Norte – UFRN – Natal (RN), Brasil.; 4 Departamento de Medicina Integrada, Universidade Federal do Rio Grande do Norte – UFRN – Natal (RN), Brasil.

**Keywords:** Multiple Sclerosis, Dizziness, Vertigo, Vestibulo-Ocular Reflex, Vestibular Function Test

## Abstract

Multiple sclerosis (MS) is a chronic and inflammatory autoimmune disease that affects the central nervous system (CNS). Dysfunction of body balance is also a common symptom and may be related to neurological injuries resulting from this disease. The aim of this study was to characterize the neurological and vestibular findings of three clinical cases diagnosed with MS. Data on the neurological evaluation and the magnetic resonance imaging of the skull were collected from the medical records. The patients responded to an initial interview and underwent clinical assessment of body balance and Video Head Impulse Test (vHIT). Vestibular symptoms and alterations were observed in at least one of the clinical tests of body balance and cerebellar function. In vHIT, changes were obtained in oculomotor tests, such as the presence of semi-spontaneous nystagmus and in parameters of the saccade test, and reduced gain in one or more vertical channels. Lesions were found on MRI of the skull in central areas that process vestibular information, such as the cerebellum and brainstem. The association of these findings suggests the presence of central vestibular dysfunction, compatible with the lesions detected in imaging exams.

## 1. INTRODUCTION

Multiple sclerosis (MS) is a chronic and inflammatory autoimmune disease that affects the central nervous system (CNS). It often affects adults between the ages of 18 and 55, but there have been exceptions^(1)^.

Although its etiology remains unknown, it is known to afflict the white matter in the form of lesions due destruction of the myelin sheath of neurons, thus leading to a deficiency in motor conduction. It is manifested by outbreaks or acute involvement that can go into remission spontaneously or through drug treatment^(1)^.

Neurological evaluation and neuroimaging tests, such as cranial magnetic resonance imaging (MRI), are necessary for etiological elucidation, as they allow, when analyzed together, to identify anatomical changes in the CNS and also in areas responsible for body balance, since vestibular dysfunction is common in this population^(2)^.

In individuals with MS, this dysfunction may be related to neurological lesions resulting from the disease, since these affect structures involved in the relay and processing of vestibular signals, that is, structures that make up the central vestibular system. Thus, it is essential to perform tests that evaluate vestibular function and its interrelation with the other systems involved in body balance.

The present study aims to characterize the neurological and vestibular findings of three clinical cases diagnosed with MS.

### Presentation of clinical cases

This study was approved by the Research Ethics Committee of Hospital Universitário Onofre Lopes – Universidade Federal do Rio Grande do Norte (CEP-HUOL-UFRN), under number 3,547,028. The three patients, from the neurology Outpatient Clinic of HUOL, signed the Informed Consent Form.

Patients diagnosed with MS according to McDonald's criteria^(3)^, answered an initial interview, in November 2019, with personal identification data related to the disease and the medicines used. These had recent MRI and neurological evaluation, performed at least one month before the vestibular evaluation (November 2019), and the data were collected from the medical records. In the imaging examination, the brain structures and nearby tissues were observed, according to the data present in the neuroradiological report. In the neurological evaluation, the following information was collected: clinical form of MS (isolated clinical syndrome, relapse-remitting, secondary progressive and primary progressive) and classification of the current state (active or non-active)^(3)^.

The patients underwent clinical evaluation of body balance and vHIT. Clinical evaluation was performed to triage vestibular function and relate it to vHIT results. The Dix-Hallpike maneuver was performed to investigate positioning nystagmus, the Diadochokinesis and Finger-to-nose tests were performed to screen cerebellar function, the Romberg and sensitized Romberg tests were performed to evaluate static balance, and the Unterberger-Fukuda (UFT) test was performed to evaluate dynamic balance. The last three took place with both eyes open and closed^(4)^.

Then, the patients underwent instrumental vestibular evaluation por middle of the device *ICS Impulse* and *OTOsuite Vestibular Software*, from the brand *Otometrics*. With the oculomotor module integrated to the vHIT equipment, the following oculomotor evaluation procedures were initially performed: research of spontaneous and semi-spontaneous nystagmus, the test of the horizontal VOR with (*Visually Enhanced Vestibulo Ocular-Reflex* - VVOR) and without (*Vestibulo Ocular Reflex Suppression* - VORS) visual optimization and research of saccadic movements^(5)^.

The VVOR and VORS have the same performance process as the cephalic impulse test, but the frequency of cephalic movement is lower (0.5 Hz). In VVOR, patients with unilateral vestibular hypofunction exhibit compensatory saccades in the same direction as the rapid phase of nystagmus, when the head turns to the affected side. When the involvement is bilateral, saccades are displayed on both sides, in the opposite direction to the movement of the head. In VORS, individuals with unilateral vestibular hypofunction exhibit larger corrective saccades to the healthy side when the head is moved to this side, while patients with bilateral vestibular hypofunction do not have saccades during head thrust to either side^(6)^.

The parameters evaluated in the saccade test were accuracy, latency and speed. Precision measures the accuracy of the eye movement divided by the path of the target, considered normal the value between 60 and 110% for the right side and 70 and 120% for the left. Latency measures the reaction time between the movement of the target and the first movement of the eye, greater than 108°/s, considered normal the value between 0 and 270ms. The speed measures the maximum agility achieved during 18.75 ms, with a value considered normal depending on the quadrant, being the right, from 0 to 10 between 0 and 250 ms and from 10 to 20 between 250 and 360 ms and the left, from -10 to 0 between 0 and 250 ms and from -10 to -20 between 250 and 360 ms^(5)^.

Then, the cephalic impulse test was performed, recorded at high frequencies (3-5Hz), starting with individual prior calibration and then applying unpredictable directional cephalic movements, low amplitude (10-20°), high acceleration (1,000-2,500°/s2) and speed (100-250°/s) in the patient for a few minutes^(5)^.

The *Otosuite Vestibular software* tracks and analyzes eye movement in reference to head movement, allows to identify compensatory saccades during (covert) and after (evident) cephalic movement and quantify, in an objective and non-invasive way, the VOR gain for each SCC individually^(5)^.

In relation to the reference values, we considered the VOR gain between 0.8 and 1.20 ms for lateral canals, 0.7 and 1.20 ms for vertical canals and asymmetry between synergistic pairs less than 20%. Abnormality can be indicated by a gain of reduced VOR and/or presence of compensatory saccades^(7)^.

#### Case 1

Patient C. O. M. S, female, 29 years and 7 months old. Presented with a clinical form of relapse-remitting and *status* not active of the disease. 3 were presented outbreaks, with the first in 2017. The medicine used was Natalizumab.

Reported complaint of vertigo lasting minutes, sporadic onset and abrupt onset. This vestibular symptom started after the first outbreak of the disease and specific treatment was never performed. She also described tinnitus episodes lasting minutes and with acute frequency.

The MRI identified ventricular system situation, morphology and normal dimensions. There was no evidence of intracranial expansive processes or extra-axial collections. Multiple and sparse hypersignal foci at T2 (TSE and FLAIR) and hyposignal at T1 (TSE) were observed, with no expansive effect or contrast medium uptake, located in the periventricular deep white matter, corona radiata, semioval centers, callosal-septal interface, as well as in the topography of the pons, bulb and cerebellar hemispheres, compatible with areas of demyelination. The rest of the white and gray substances showed normal signal intensities. There were no areas of restricted diffusion, and the nuclei of the base, thalamus, cortical grooves and cisterns maintained their anatomical appearance. Hippocampus with normal morphology, dimensions and signal strength. There was no anomalous impregnation by the paramagnetic agent.

It was verified lateropulsion to both sides in the Romberg and sensitized Romberg tests. The patient was unable to perform the UFT. Dysmetria in the Finger-to-nose and dysdiadochokinesia were observed. The Dix-Hallpike maneuver was negative bilaterally.

As for vHIT, in oculomotor tests, the absence of spontaneous nystagmus and the presence of semi-spontaneous nystagmus were observed (*gaze*) bidirectional horizontal, which hit according to the direction of the evaluated gaze.

The VVOR was within normal standards, while the VORS was altered. The compensatory saccades of greater amplitude verified in this examination suggest asymmetry of vestibular response, with the left side considered hypoactive in relation to the right, because it presents saccades of lower amplitude.

The saccade test, considering the average of the responses, showed accuracy (97% to the right and 85% to the left), speed (219°/s) and latency to the right (283ms) adequate, but speed to the left (-136º/s) decreased and latency to the left (366ms) increased. It was also observed the presence of hypermetry, that is, saccades with greater amplitude in relation to the target, as a qualitative finding regarding accuracy.

**Figure 1:**
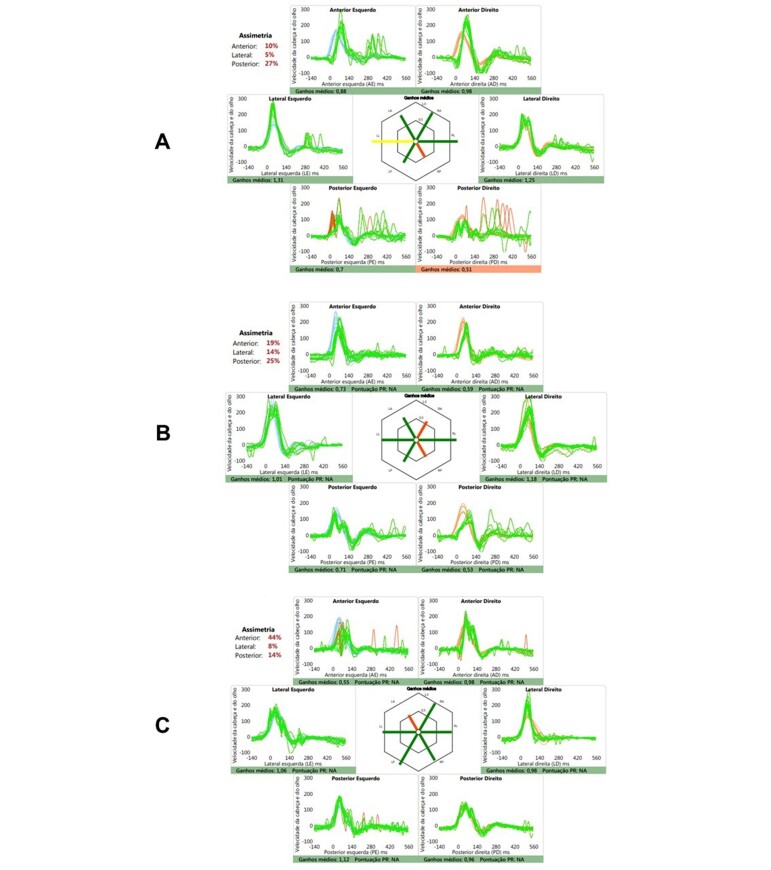
Video Head Impulse Test recordings after head impulses in the plane of each semicircular canal in both ears of cases 1 (A), 2 (B) and 3 (C). In blue and pink is the speed of the head corresponding to the left and right sides, respectively. In green is the speed of the eye equivalent to each impulse of the head. In the central hexagonal graph is the gain value of the answers: in red, altered results, and in green, normal results. In red, the occurrence of compensatory saccades is noted.

In the video head impulse test, reduced VOR gain was obtained for the right posterior SCC, increased VOR gain for the left lateral SCC, and increased asymmetry between the posterior canals (>20%) (Figure 1).

Assimetria = Asymmetry; Anterior = Anterior; Lateral = Lateral; Posterior = Posterior; Ganhos médios = Mean gain; Lateral Esquerdo = Left Lateral (LL); Lateral Direito = Right Lateral (RL); Posterior Esquerdo = Left Posterior (LP); Posterior Direito = Right Posterior (RP); Anterior Esquerdo = Left Anterior (LA); Anterior Direito = Right Anterior (RA); Velocidade da cabeça e do olho = Head and eye velocity; Pontuação PR: NA (ausente) = Score PR:NA (absent).

It was also verified the presence of hidden and evident compensatory saccades in the right posterior canal, affected by the deficit gain. In the left posterior SCC, saccade-like movements were also observed, but with a lower latency than the appearance of the covered saccades, suggestive of the presence of artifacts, also compatible with the slowing of saccade movements when looking to the left (Figure 1).

#### Case 2

Patient D.A.S., female, aged 36 years and 10 months. The clinical form was secondary progressive MS, with not active disease *status*. She had 3 outbreaks, the first in 2001. The use of the drug Natalizumab has been described.

She complained of body imbalance, starting five years ago and irregular appearance, being accompanied by physiotherapeutic intervention. She also reported worsening of this condition in recent months, given the progressive nature of MS.

MRI showed multiple lesions in the subcortical and deep white matter, with predominance in periventricular regions, semioval centers, U-fibers of the frontal lobes, some parallel to the septal callus interface and others coalescing, nonspecific, probably related to demyelinating substrate lesions. Rare lesions of the *black holes* type in semioval centers. Demyelinating plaques with post-contrast enhancement, infratentorial lesions or new lesions were not characterized. Atrophy of the corpus callosum, cerebellum, brain stem and accentuation of the cerebral sulci and cisterns with compensatory ectasia of the supratentorial ventricular system were observed, inferring brain volumetric reduction. There was no evidence of intraparenchymal expansive processes, nor of supratentorial or infratentorial extra-axial collections. The diffusion sequence showed no restriction area, centered fourth ventricle with conserved morphology and centered median structures.

In the evaluation of static and dynamic equilibrium, the Romberg test showed no changes and in the sensitized Romberg, instability was observed. The patient was unable to perform the UFT. As for the screening tests of cerebellar function, dysdiadochokinesia was verified, while the Finger-to-nose was normal. The Dix-Hallpike maneuver was negative, bilaterally.

In the vHIT, referring to oculomotor tests, it was observed absence of spontaneous nystagmus and presence of semi-spontaneous nystagmus (*gaze*) horizontal to the left. The saccade test showed mean adequate responses for accuracy (90% to the right and 102% to the left) and speed (199°/s to the right and-263º/s to the left), but latency with a slight increase bilaterally (271ms to the right and 272ms to the left). In VVOR and VORS, no changes were observed.

The video head impulse test showed decreased VOR gain for the right anterior and posterior SCCs, as well as increased asymmetry between the posterior canals. In this case, the occurrence of any type of compensatory saccade was not observed (Figure 1).

#### Case 3

Patient A.C.S, female, aged 39 years and 10 months. The MS presented was of the relapse-remitting clinical form, with not active disease *status*. It manifested only one outbreak in the year 2019. The medicine used was Tecfidera.

Described vertigo and presence of tinnitus in both ears. Reported that these vestibular symptoms began after the first outbreak of the disease.

MRI examination did not observe infra-or supratentorial expansive processes. Ventricular system with normal situation, morphology and dimensions. Presence of multiple oval-shaped lesions with hypersignal in T2/FLAIR distributed in the periventricular cerebral white matter, with involvement of the corpus callosum trunk, without expansive effect or uptake of contrast medium. There was no restriction to the diffusion of water molecules in the brain parenchyma. Sulcus, fissures and cisterns of anatomical appearance. Craniocervical transition without abnormalities.

No changes were observed in the static and dynamic equilibrium tests. The UFT was altered, and an angular deviation greater than 45º to the left was observed.

The Diadochokinesis and Finger-to-nose tests were unchanged. The Dix-Hallpike maneuver was negative bilaterally.

As for the oculomotor tests of vHIT, the absence of spontaneous and semi-spontaneous nystagmus was observed (*gaze*). In the saccade test, it presented accuracy (88% to the right and 101% to the left) and latency (206ms to the right and 222ms to the left) within the standards of normality and speed (293º/s to the right and -362º/s to the left) increased. The results of VVOR and VORS showed no changes.

In the video cephalic impulse test, there was a deficit of VOR gain for the left anterior canal, as well as an increase in asymmetry between the anterior canals (>20%) (Figure 1).

The occurrence of hidden and evident compensatory saccades in the left anterior canal and, only evident, in the right anterior and left posterior canals was verified (Figure 1).

Chart 1 summarizes the three clinical cases described above.

**Chart 1:** Summary of neurological and vestibular findings in the three clinical cases of Multiple Sclerosis described

**Data**	**Case 1**	**Case 2**	**Case 3**
**Personal identification**	Female	Female	Female
	27 years and 7 months old	36 years and 10 months old	39 years and 10 months old
**Medication used for MS**	Natalizumab	Natalizumab	Tecfidera
**Otoneurological symptoms**	Vertigo lasting minutes	Body imbalance	Vertigo
	High-pitched tinnitus lasting minutes		Bilateral tinnitus
**Neurological assessment**	MS non-active relapse-remitting	Secondary progressive MS not active	MS non-active relapse-remitting
	3 outbreaks (first in 2017)	3 outbreaks (first in 2001)	1 outbreak in 2019
**MRI of the skull**	Alteration in brain stem and cerebellar regions	Alteration in brain stem and cerebellar regions	Absence of changes in brain stem and cerebellar regions
**Clinical assessment of body balance**	Lateropulsion for both sides in the Romberg and sensitized Romberg tests	Romberg unchanged	Romberg and sensitized Romberg unchanged
	Failed to perform the UFT	Romberg sensitized with instability	Altered UFT (angular deviation greater than 45º to the left)
	Finger-to-nose dysmetria	Failed to perform the UFT	Diadochokinesia and unchanged Finger-to-nose
	Dysdiadochokinesia	Dysdiadochokinesia	Dix-Hallpike negative bilaterally
	Dix-Hallpike negative bilaterally	Finger-to-nose normal	
		Dix-Hallpike negative bilaterally	
**vHIT oculomotor tests**	Absent spontaneous nystagmus	Absent spontaneous nystagmus	Absent spontaneous and semi-spontaneous nystagmus
	Presence of bidirectional horizontal semi-spontaneous nystagmus (beat according to gaze direction)	Presence of horizontal semi-spontaneous nystagmus to the left	VVOR and VORS within the standards of normality
	VVOR within normal standards	VVOR and VORS within the standards of normality	Saccade testing with adequate accuracy and latency and bilaterally increased speed
	Altered VORS with vestibular response asymmetry	Saccade testing with adequate accuracy and speed and bilaterally increased latency	
	Saccadic test with decreased left speed and increased left latency		
	Presence of hypermetry		
**vHIT head impulse test**	Reduced gain with VOR for right posterior SCC	VOR gain decreased for right anterior and posterior SCCs	Decreased VOR gain for left anterior SCC
	Increased VOR gain for left side SCC	Increased asymmetry between posterior SCCs	Increased asymmetry between previous SCCs
	Increased asymmetry between posterior canals	No compensatory saccades occurred	Presence of compensatory saccades covered and evident in the anterior left SCC and evident in the anterior right and posterior left SCCs
	Presence of concealed and evident compensatory saccades in the right posterior SCC		

**Caption:** vHIT - video head impulse test; MS - multiple sclerosis; UFT - unterberger-fukuda test; VVOR - vestibulo-ocular reflex with visual optimization; VORS - vestibulo-ocular reflex without visual optimization; VOR - vestibulo-ocular reflex; SCC - semicircular canal; SCCs semicircular canals.

## DISCUSSION

In the present study, two patients presented vertigo and one presented body imbalance. Vertigo may be the overt symptom of MS in 4-20% of patients. It is checked regularly as a preliminary manifestation to medical diagnosis^(8)^.

Body imbalance is also a frequent symptom in this population. It can be caused by the involvement of brain areas of the posterior cranial fossa, such as the brainstem and cerebellum, due to the presence of typical demyelinating foci of the disease^(8)^.

Changes were verified in the clinical tests of static and dynamic body balance, differently between the samples, such as lateropulsion to both sides, instability and altered UFT or inability to perform it. The findings regarding the Romberg test are consistent with the description of a clinical case with MS^(9)^.

In the UFT, there was an angular deviation greater than 45º to the left, same side with decreased gain of the previous SCC, in case 3. The side of the deviation corresponds to the same side of the hypofunction and this can be justified by the exit of the spinal vestibule pathway, since it exerts ipsilateral tonic influence on the muscles acting on the lower limbs^(10)^. Cases 1 and 2 failed to perform the test, which corresponds to a more pronounced impairment related to dynamic balance than the alteration described previously.

Regarding cerebellar function, the tests showed the presence of dysmetria in the Finger-to-nose in case 1 and dysdiadochokinesia in cases 1 and 2. Both patients presented impairment of cerebellar areas, which may justify such findings in the tests.

Case 1 presented bidirectional horizontal semi-spontaneous nystagmus, according to change of gaze direction, while case 2 presented horizontal semi-spontaneous nystagmus to the left.

The presence of semi-spontaneous nystagmus is verified in cerebellar lesions, compatible with the cases in question. This nystagmus is present in 20-80% of MS cases and occurs due to defect in the integrative neural network, structures that encompass the cerebellum, which causes inability to maintain gaze in certain positions^(11)^.

As for bidirectional nystagmus, a study^(11)^ recorded the presence in four patients, being horizontal in three cases and oblique in one, compatible with case 1 of this study. The presence of this type of nystagmus is a relevant data for topodiagnosis, since its occurrence in more than one position of the gaze is always of central origin.

In the saccade test, all cases showed changes in speed and/or latency parameters. A study^(11)^ that evaluated 30 individuals with MS found abnormality in all individuals, in at least one of the parameters studied, being 96.7% in speed, 20% in accuracy and 6.7% in latency.

Discrete changes in these parameters may be present in patients with peripheral vestibular dysfunctions. However, in case 1 it was verified alteration of the morphology of the trace, the saccadic dysmetria of the hypermetric type, saccadic movement that ends below the target, suggestive of changes in the cerebellum and in the brain stem^(12)^. The patient in this study had lesions in both structures.

The VORS, altered in case 1, was suggestive of vestibular hypofunction on the left, compatible with a decrease in the speed of saccadic movements on the left. In addition, the cerebellum is important for the generation of VORS, especially the cerebellar *vermis*, which makes this test relevant in neurological lesions^(6)^. Case 1 presented involvement of the cerebellar region, which is in accordance with the scientific literature.

It was observed, in the head impulse test, a predominance of reduced VOR gain of the vertical SCCs in relation to the lateral ones in the three cases described. This pattern is not characteristic of peripheral vestibulopathy^(13)^, which thus represents impairment of the central vestibular pathways.

There are three distinct patterns of involvement of SCCs in acute unilateral vestibulopathy according to innervation: hypofunction of the anterior and lateral canals or only lateral, due to injury to the superior vestibular nerve; hypofunction of all canals, due to injury to the superior and inferior vestibular nerves; and hypofunction of the posterior canal, due to injury to the inferior vestibular nerve. Thus, when damage occurs to the labyrinth or any of the branches of the vestibular nerve, a unique combination of deficits is expected. Thus, restricted hypofunction of the vertical channels (case 2) or only of the anterior (case 3) do not fit as exclusively peripheral impairment, according to this criterion^(13)^.

Although case 1 does not fit this aforementioned standard, a study^(14)^ described the isolated involvement of one or both posterior canals as a result of damage to the brain stem, compatible with the MRI examination of the case in question.

In case 1, an increased gain in the left lateral SCC was observed. Considering that there was no deficient calibration or excessive proximity to the target and that the patient underwent retesting and maintained the results, the increase in gain may be related to possible hypersensitivity of the vestibular system, which is the abnormal perception of head movement. This ability is often compromised in core alterations^(15)^.

Regarding compensatory saccades, these were seen only in vertical canals, both covered and evident, not only in canals with reduced VOR gain, but also with gain within normal standards. A study^(2)^ also observed the presence of saccades in individuals with MS.

The high incidence of signs of vestibular involvement in individuals with MS of central origin indicates the need for investigation of this system in the diagnostic orientation of this disease. Vestibular dysfunction usually originates from the combination of various impairments, making it difficult to establish its causes. Body balance can be affected by the involvement of one or more sensorimotor systems that contribute to its maintenance, due to the diffuse locations of neurological lesions. In this way, it is important that several tests are carried out, in association, to evaluate the body balance of this population.

The association of the findings presented suggests the presence of central vestibular dysfunction, since the alterations found in the clinical and oculomotor tests performed, such as lateropulsion and instability present in the Romberg and sensitized Romberg test, alteration in the saccade test, dysmetria, dysdiadochokinesia, presence of bidirectional semi-spontaneous nystagmus, hypermetric saccades, alteration of the VORS and gain of the deficient VOR restricted to vertical SCCs, characterize findings compatible with central disorder.

As evidenced by the results and studies discussed here, vestibular dysfunction may be present in MS, since demyelinating lesions may involve areas that participate in the central processing of vestibular information. These areas integrate and interpret the sensory information that reaches the CNS^(8)^. Thus, individuals with this disease deserve special attention regarding the vestibular evaluation and body balance, as it will direct the choice of the best treatment in view of the signs present in each case.

The present study highlights the need to expand the research with a larger sample size to allow statistical inferences. It is also suggested that new studies can evaluate individuals with different MS phenotypes, aiming to characterize the specific findings of each clinical form and also employ other instruments to investigate the different systems that control body balance.

## FINAL COMMENTS

The association of the findings obtained in the clinical, oculomotor tests and in the video head impulse test, together with the lesions evidenced in the MRI in central areas that process vestibular information, such as cerebellum and brainstem, suggest the presence of central vestibular dysfunction.
