# Supplementary material for: Neurological and vestibular findings in three cases of Multiple Sclerosis
Source: Codas. 2023 Nov 20;35(6):e20210153. doi: 10.1590/2317-1782/20232021153en (PMC10702714; doi:10.1590/2317-1782/20232021153en)
Supplement: Translation

# Translation: Achados neurológicos e vestibulares em três casos de Esclerose Múltipla

**DOI:** 10.1590/2317-1782/20232021153pt

**Authors:** Alanna Stefany de Lima Evangelista, José Diniz, Ana Paula Machado Costa, Mário Emílio Teixeira Dourado, Erika Barioni Mantello

**Affiliations:** 1 Programa Associado de Pós-graduação em Fonoaudiologia, Universidade Federal do Rio Grande do Norte – UFRN – Natal (RN), Brasil.; 2 Departamento de Cirurgia, Universidade Federal do Rio Grande do Norte – UFRN – Natal (RN), Brasil.; 3 Hospital Universitário Onofre Lopes, Universidade Federal do Rio Grande do Norte – UFRN – Natal (RN), Brasil.; 4 Departamento de Medicina Integrada, Universidade Federal do Rio Grande do Norte – UFRN – Natal (RN), Brasil.

**Keywords:** Esclerose Múltipla, Tontura, Vertigem, Reflexo Vestíbulo-Ocular, Testes de Função Vestibular

## Abstract

A Esclerose Múltipla (EM) é uma doença autoimune crônica e inflamatória que afeta o sistema nervoso central (SNC). A disfunção do equilíbrio corporal também é um sintoma comum e pode estar relacionada às lesões neurológicas decorrentes desta doença. O objetivo deste estudo foi caracterizar os achados neurológicos e vestibulares de três casos clínicos com diagnóstico de EM. Dados sobre a avaliação neurológica e do exame de ressonância magnética de crânio foram coletados dos prontuários. Os pacientes responderam a uma entrevista inicial e foram submetidos à avaliação clínica do equilíbrio corporal e ao *Video Head Impulse Test* (vHIT). Observou-se presença de sintomas vestibulares e alterações em pelo menos uma das provas clínicas do equilíbrio corporal e função cerebelar. No vHIT, obteve-se alterações em testes oculomotores, como presença de nistagmo semi-espontâneo e em parâmetros do teste sacádico, e ganho reduzido em um ou mais canais verticais. Foram verificadas lesões, na ressonância magnética de crânio, em áreas centrais que processam as informações vestibulares, como cerebelo e tronco encefálico. A associação destes achados sugere a presença de disfunção vestibular central, compatível com as lesões detectadas nos exames de imagem.

## INTRODUÇÃO

A Esclerose Múltipla (EM) é uma doença autoimune crônica e inflamatória que afeta o sistema nervoso central (SNC). Atinge, frequentemente, adultos na faixa de 18 a 55 anos de idade, mas exceções têm ocorrido^(1)^.

Embora a sua etiologia permaneça desconhecida, sabe-se que aflige a substância branca em forma de lesões devido destruição da bainha de mielina dos neurônios, levando, assim, a uma deficiência na condução motora. Manifesta-se por surtos ou acometimentos agudos que podem entrar em remissão espontaneamente ou por meio de tratamento medicamentoso^(1)^.

A avaliação neurológica e os exames de neuroimagem, como a ressonância magnética de crânio (RMC), são necessários para elucidação etiológica, pois permitem, quando analisados em conjunto, identificar alterações anatômicas do SNC e, também, em áreas responsáveis pelo equilíbrio corporal, uma vez que a disfunção vestibular é comum nessa população^(2)^.

Em indivíduos com EM, essa disfunção pode estar relacionada às lesões neurológicas decorrentes da doença, uma vez que estas afetam estruturas envolvidas na retransmissão e no processamento dos sinais vestibulares, ou seja, estruturas que compõem o sistema vestibular central. Dessa forma, é fundamental a realização de testes que avaliem a função vestibular e sua inter-relação com os demais sistemas envolvidos no equilíbrio corporal.

O presente estudo objetiva caracterizar os achados neurológicos e vestibulares de três casos clínicos com diagnóstico de EM.

### Apresentação dos casos clínicos

Este estudo foi aprovado pelo Comitê de Ética em Pesquisa do Hospital Universitário Onofre Lopes – Universidade Federal do Rio Grande do Norte (CEP-HUOL-UFRN), sob o número 3.547.028. Os três pacientes, provenientes do Ambulatório de Neurologia do HUOL, assinaram o Termo de Consentimento Livre e Esclarecido.

Os pacientes, diagnosticados com EM de acordo com os critérios de McDonald^(3)^, responderam uma entrevista inicial, em novembro de 2019, com dados de identificação pessoal, relacionados à doença e aos medicamentos utilizados. Estes apresentavam RMC e avaliação neurológica recentes, realizadas há pelo menos um mês antes da avaliação vestibular (novembro de 2019), sendo os dados coletados dos prontuários. No exame de imagem, observaram-se as estruturas encefálicas e os tecidos próximos, de acordo com os dados presentes no laudo neurorradiológico. Na avaliação neurológica, levantaram-se as seguintes informações: forma clínica da EM (síndrome clínica isolada, surto-remissão, secundariamente progressiva e primariamente progressiva) e classificação do estado atual (ativa ou não ativa)^(3)^.

Os pacientes foram submetidos à avaliação clínica do equilíbrio corporal e ao vHIT. A avaliação clínica foi realizada para triar a função vestibular e relacioná-la aos resultados do vHIT. Realizou-se a manobra de Dix-Hallpike para a pesquisa do nistagmo de posicionamento, as provas de Diadococinesia e *Index-Naso* para a triagem da função cerebelar, as provas de Romberg e Romberg sensibilizado para avaliação do equilíbrio estático e a prova de Unterberger-Fukuda (PUF) para avaliação do equilíbrio dinâmico. Estas três últimas efetuaram-se tanto com os olhos abertos como fechados^(4)^.

Em seguida, os pacientes submeteram-se à avaliação vestibular instrumental por meio do dispositivo *ICS Impulse* e *software OTOsuite Vestibular*, da marca *Otometrics*. Com o módulo oculomotor integrado ao equipamento vHIT, realizaram-se, inicialmente, os procedimentos de avaliação oculomotora: a pesquisa do nistagmo espontâneo e semi-espontâneo, o teste do RVO horizontal com (*Visually Enhanced Vestibulo Ocular Reflex* - VVOR) e sem (*Vestibulo Ocular Reflex Suppression* - VORS) otimização visual e a pesquisa dos movimentos sacádicos^(5)^.

O VVOR e VORS apresentam o mesmo processo de realização que o teste de impulso cefálico, porém a frequência da movimentação cefálica é inferior (0.5Hz). No VVOR, pacientes com hipofunção vestibular unilateral exibem sacadas compensatórias na mesma direção da fase rápida do nistagmo, quando a cabeça gira para o lado afetado. Quando o acometimento é bilateral, são exibidas sacadas em ambos os lados, na direção oposta ao movimento da cabeça. No VORS, os indivíduos com hipofunção vestibular unilateral exibem sacadas corretivas maiores para o lado saudável, quando a cabeça é movida para este lado, enquanto que os pacientes com hipofunção vestibular bilateral não apresentam sacadas durante o impulso de cabeça para nenhum dos lados^(6)^.

Os parâmetros avaliados no teste sacádico foram precisão, latência e velocidade. A precisão mede a exatidão do movimento do olho dividida pelo percurso do alvo, considerado normal o valor entre 60 e 110% para o lado direito e 70 e 120% para o esquerdo. A latência mede o tempo de reação entre o movimento do alvo e o primeiro movimento do olho, maior que 108°/s, considerada normal o valor entre 0 e 270 ms. A velocidade mede a agilidade máxima alcançada durante 18,75 ms, com valor considerado normal dependendo do quadrante, sendo o direito, de 0 a 10 entre 0 e 250 ms e de 10 a 20 entre 250 e 360 ms e, o esquerdo, de -10 a 0 entre 0 e 250 ms e de -10 a -20 entre 250 e 360 ms^(5)^.

Em seguida, realizou-se o teste do impulso cefálico, registrado em altas frequências (3-5 Hz), iniciando pela calibração prévia individual e depois pela aplicação de movimentos cefálicos de direção imprevisíveis, baixa amplitude (10-20°), alta aceleração (1.000-2.500°/s^2^) e velocidade (100-250°/s) no paciente por alguns minutos^(5)^.

O *software OTOsuite Vestibular* rastreia e analisa o movimento dos olhos em referência ao movimento da cabeça, permite identificar sacadas compensatórias durante (encobertas) e após (evidentes) o movimento cefálico e quantificar, de forma objetiva e não invasiva, o ganho do RVO para cada CSC individualmente^(5)^.

Em relação aos valores de referência, foi considerado o ganho do RVO entre 0,8 e 1,20 ms para os canais laterais, 0,7 e 1,20 ms para os verticais e assimetria entre os pares sinérgicos menor que 20%. A anormalidade pode ser indicada por um ganho do RVO reduzido e/ou presença de sacadas compensatórias^(7)^.

#### Caso 1

Paciente C.O.M.S, do sexo feminino, com 29 anos e 7 meses de idade. Apresentava EM com forma clínica surto-remissão e *status* não ativo da doença. Apresentou 3 surtos, com o primeiro em 2017. O medicamento utilizado era o Natalizumabe.

Relatou queixa de vertigem com duração de minutos, aparecimento esporádico e surgimento abrupto. Este sintoma vestibular iniciou após o primeiro surto da doença e nunca foi realizado tratamento específico. Descreveu, ainda, episódios de zumbido com duração de minutos e frequência aguda.

A RMC identificou sistema ventricular de situação, morfologia e dimensões normais. Não houve evidências de processos expansivos intracranianos ou de coleções extra-axiais. Observou-se presença de múltiplos e esparsos focos de hipersinal em T2 (TSE e FLAIR) e hipossinal em T1 (TSE), sem efeito expansivo ou captação do meio de contraste, localizados na substância branca profunda periventricular, coroas radiadas, centros semi-ovais, interface caloso-septal, bem como em topografia da ponte, bulbo e dos hemisférios cerebelares, compatíveis com áreas de desmielinização. O restante das substâncias branca e cinzenta apresentaram intensidades de sinal normais. Não se verificou áreas de restrição à difusão, bem como os núcleos da base, tálamo, sulcos corticais e cisternas mantiveram seu aspecto anatômico. Hipocampo com morfologia, dimensões e intensidade de sinal normais. Não houve impregnação anômala pelo agente paramagnético.

Foi verificado lateropulsão para ambos os lados nas provas de Romberg e Romberg sensibilizado. A paciente não conseguiu realizar a PUF. Observou-se dismetria na *Index-Naso* e disdiadococinesia. A manobra de Dix-Hallpike foi negativa bilateralmente.

Quanto ao vHIT, nos testes oculomotores, observou-se ausência de nistagmo espontâneo e presença de nistagmo semi-espontâneo (*gaze*) horizontal bidirecional, que bateu conforme direção do olhar avaliado.

O VVOR estava dentro dos padrões de normalidade, enquanto o VORS encontrou-se alterado. As sacadas compensatórias de maior amplitude verificadas neste exame sugerem assimetria de resposta vestibular, sendo o lado esquerdo considerado hipoativo em relação ao direito, por apresentar sacadas de menor amplitude.

O teste sacádico, considerando a média das respostas, apresentou precisão (97% para a direita e 85% para a esquerda), velocidade (219°/s) e latência para a direita (283 ms) adequadas, mas velocidade para a esquerda (-136º/s) diminuída e latência para a esquerda (366 ms) aumentada. Foi observada, ainda, presença de hipermetria, ou seja, sacádicos com maior amplitude em relação ao alvo, como achado qualitativo referente à precisão.

No teste de impulso cefálico por vídeo foi obtido ganho reduzido do RVO para o CSC posterior direito, ganho aumentado do RVO para o CSC lateral esquerdo e aumento da assimetria entre os canais posteriores (>20%) (Figura 1).

**Figura 1:** Registros do exame *Video Head Impulse Test*. O após impulsos cefálicos no plano de cada canal semicircular em ambas as orelhas dos casos 1 (A), 2 (B) e 3 (C). Em azul e rosa está a velocidade da cabeça correspondente aos lados esquerdo e direito, respectivamente. Em verde está a velocidade do olho equivalente a cada impulso da cabeça. No gráfico hexagonal central encontra-se o valor de ganho das respostas: em vermelho, resultados alterados, e em verde, resultados normais. Em vermelho, observa-se a ocorrência de sacadas compensatórias.

Foi também verificada a presença de sacadas compensatórias encobertas e evidentes no canal posterior direito, afetado pelo ganho deficitário. Observou-se, ainda, no CSC posterior esquerdo, movimentos semelhantes a sacadas, porém numa latência inferior ao surgimento das sacadas encobertas, sugestivo de presença de artefatos, compatível também com a lentificação dos movimentos sacádicos ao olhar para a esquerda (Figura 1).

#### Caso 2

Paciente D.A.S., do sexo feminino, com 36 anos e 10 meses de idade. A forma clínica da EM era secundariamente progressiva, com *status* não ativo da doença. Apresentou 3 surtos, com o primeiro em 2001. Foi descrito o uso do fármaco Natalizumabe.

Queixou-se de desequilíbrio corporal, com início há cinco anos e de aparecimento irregular, sendo acompanhada por intervenção fisioterapêutica. Relatou, ainda, piora desse quadro nos últimos meses, tendo em vista o caráter progressivo da EM.

O exame de RMC demonstrou múltiplas lesões na substância branca subcortical e profunda, com predomínio em regiões periventriculares, centros semiovais, fibras em U dos lobos frontais, sendo algumas paralelas a interface caloso septal e outras coalescentes, inespecíficas, provavelmente relacionadas às lesões de substrato desmielinizante. Foram verificadas raras lesões do tipo *black holes* em centros semiovais. Não foram caracterizadas placas desmielinizantes com realce pós-contraste, nem lesões infratentoriais ou novas lesões. Foi observada atrofia do corpo caloso, cerebelo, tronco encefálico e acentuação dos sulcos e cisternas cerebrais com ectasia compensatória do sistema ventricular supratentorial, inferindo redução volumétrica encefálica. Não houve evidência de processos expansivos intraparenquimatosos, nem de coleções extra-axiais supratentoriais ou infratentoriais. A sequência de difusão não evidenciou área de restrição, quarto ventrículo centrado e com morfologia conservada e estruturas medianas centradas.

Na avaliação do equilíbrio estático e dinâmico, a prova de Romberg não apresentou alterações e na Romberg sensibilizado, observou-se instabilidade. A paciente não conseguiu realizar a PUF. Quanto às provas de triagem da função cerebelar, foi verificada disdiadococinesia, enquanto a *Index-Naso* esteve normal. A manobra de Dix-Hallpike foi negativa, bilateralmente.

No vHIT, referente aos testes oculomotores, observou-se ausência de nistagmo espontâneo e presença de nistagmo semi-espontâneo (*gaze*) horizontal para a esquerda. O teste sacádico exibiu média de respostas adequadas para precisão (90% para a direita e 102% para a esquerda) e velocidade (199°/s para a direita e -263º/s para a esquerda), porém latência com discreto aumento bilateralmente (271 ms para a direita e 272 ms para a esquerda). No VVOR e VORS, não foram observadas alterações.

O teste do impulso cefálico por vídeo evidenciou ganho do RVO diminuído para os CSCs anterior e posterior direito, como também aumento da assimetria entre os canais posteriores. Não foi observada, neste caso, a ocorrência de nenhum tipo de sacada compensatória (Figura 1).

#### Caso 3

Paciente A.C.S, do sexo feminino, com 39 anos e 10 meses de idade. A EM apresentada era da forma clínica surto-remissão, com *status* não ativo da doença. Manifestou apenas um surto no ano de 2019. O medicamento utilizado era o Tecfidera.

Descreveu vertigem e presença de zumbido em ambas as orelhas. Relatou que esses sintomas vestibulares iniciaram após o primeiro surto da doença.

O exame de RMC não observou processos expansivos infra ou supratentoriais. Sistema ventricular com situação, morfologia e dimensões normais. Presença de múltiplas lesões ovaladas com hipersinal em T2/FLAIR distribuídas na substância branca cerebral periventricular, com envolvimento do tronco do corpo caloso, sem efeito expansivo ou captação do meio de contraste. Não se verificou restrição à difusão das moléculas da água no parênquima encefálico. Sulcos, fissuras e cisternas de aspecto anatômico. Transição crânio-cervical sem anormalidades.

Não foram observadas alterações nas provas de equilíbrio estático e dinâmico. A PUF foi alterada, sendo observado desvio angular maior que 45º para a esquerda.

As provas Diadococinesia e *Index-Naso* apresentaram-se sem alterações. A manobra de Dix-Hallpike foi negativa bilateralmente.

Quanto aos testes oculomotores do vHIT, observou-se ausência de nistagmo espontâneo e semi-espontâneo (*gaze*). No teste sacádico, apresentou precisão (88% para a direita e 101% para a esquerda) e latência (206 ms para a direita e 222 ms para a esquerda) dentro dos padrões de normalidade e velocidade (293º/s para a direita e -362º/s para a esquerda) aumentada. Os resultados do VVOR e VORS não apresentaram alterações.

No teste de impulso cefálico por vídeo, obteve-se um ganho do RVO deficitário para o canal anterior esquerdo, como também aumento da assimetria entre os canais anteriores (>20%) (Figura 1).

Foi verificada a ocorrência de sacadas compensatórias encobertas e evidentes no canal anterior esquerdo e, apenas evidentes, nos canais anterior direito e posterior esquerdo (Figura 1).

No Quadro 1, encontra-se o resumo dos três casos clínicos descritos anteriormente.

**Quadro 1:** Resumo dos achados neurológicos e vestibulares nos três casos clínicos de Esclerose Múltipla descritos

**Dados**	**Caso 1**	**Caso 2**	**Caso 3**
**Identificação pessoal**	Sexo feminino	Sexo feminino	Sexo feminino
	27 anos e 7 meses de idade	36 anos e 10 meses de idade	39 anos e 10 meses de idade
**Medicamentos utilizados para a EM**	Natalizumabe	Natalizumabe	Tecfidera
**Sintomas otoneurológicos**	Vertigem com duração de minutos	Desequilíbrio corporal	Vertigem
	Zumbido agudo com duração de minutos		Zumbido bilateral
**Avaliação neurológica**	EM surto-remissão não ativo	EM secundariamente progressiva não ativo	EM surto-remissão não ativo
	3 surtos (primeiro em 2017)	3 surtos (primeiro em 2001)	1 surto em 2019
**Ressonância magnética de crânio**	Alteração em tronco encefálico e regiões cerebelares	Alteração em tronco encefálico e regiões cerebelares	Ausência de alterações em tronco encefálico e regiões cerebelares
**Avaliação clínica do equilíbrio corporal**	Lateropulsão para ambos os lados nas provas de Romberg e Romberg sensibilizado	Romberg sem alterações	Romberg e Romberg sensibilizado sem alterações
	Não conseguiu realizar a PUF	Romberg sensibilizado com instabilidade	PUF alterada (desvio angular maior que 45º para a esquerda)
	Dismetria na *Index-Naso*	Não conseguiu realizar a PUF	Diadococinesia e *Index-Naso* sem alterações
	Disdiadococinesia	Disdiadococinesia	Dix-Hallpike negativo bilateralmente
	Dix-Hallpike negativo bilateralmente	*Index-Naso* normal	
		Dix-Hallpike negativo bilateralmente	
**Testes oculomotores do vHIT**	Nistagmo espontâneo ausente	Nistagmo espontâneo ausente	Nistagmos espontâneo e semi-espontâneo ausentes
	Presença de nistagmo semi-espontâneo horizontal bidirecional (batimento conforme direção do olhar)	Presença de nistagmo semi-espontâneo horizontal para a esquerda	VVOR e VORS dentro dos padrões de normalidade
	VVOR dentro dos padrões de normalidade	VVOR e VORS dentro dos padrões de normalidade	Teste sacádico com precisão e latência adequadas e velocidade aumentada bilateralmente
	VORS alterado com assimetria de resposta vestibular	Teste sacádico com precisão e velocidade adequadas e latência aumentada bilateralmente	
	Teste sacádico com velocidade para a esquerda diminuída e latência para a esquerda aumentada		
	Presença de hipermetria		
**Teste do impulso cefálico do vHIT**	Ganho reduzido com RVO para o CSC posterior direito	Ganho do RVO diminuído para os CSCs anterior e posterior direito	Ganho do RVO diminuído para o CSC anterior esquerdo
	Ganho aumentado do RVO para o CSC lateral esquerdo	Aumento da assimetria entre os CSCs posteriores	Aumento da assimetria entre os CSCs anteriores
	Aumento da assimetria entre os canais posteriores	Não ocorreram sacadas compensatórias	Presença de sacadas compensatórias encobertas e evidentes no CSC anterior esquerdo e evidentes nos CSCs anterior direito e posterior esquerdo
	Presença de sacadas compensatórias encobertas e evidentes no CSC posterior direito		

**Legenda:** vHIT - *video head impulse test*; EM - esclerose múltipla; PUF - prova de unterberger-fukuda; VVOR - reflexo vestíbulo-ocular com otimização visual; VORS - reflexo vestíbulo-ocular sem otimização visual; RVO - reflexo vestíbulo-ocular; CSC - canal semicircular; CSCs canais semicirculares.

## DISCUSSÃO

No presente estudo, duas pacientes apresentaram vertigem e uma, desequilíbrio corporal. A vertigem pode ser o sintoma manifesto da EM em 4-20% dos pacientes. É verificada regularmente como uma manifestação preliminar ao diagnóstico médico^(8)^.

O desequilíbrio corporal também é um sintoma frequente nesta população. Pode ser provocado pelo comprometimento de áreas encefálicas da fossa posterior do crânio, como o tronco encefálico e cerebelo, devido presença de focos desmielinizantes típicos da doença^(8)^.

Foram verificadas alterações nos testes clínicos do equilíbrio corporal estático e dinâmico, de forma distinta entre a amostra, como lateropulsão para ambos os lados, instabilidade e PUF alterada ou inabilidade em realizá-la. Os achados em relação a Prova de Romberg são concordantes com a descrição de um caso clínico com EM^(9)^.

Na PUF, verificou-se desvio angular maior que 45º para a esquerda, mesmo lado com ganho diminuído do CSC anterior, no caso 3. O lado do desvio corresponde ao mesmo lado da hipofunção e isso pode ser justificado pela saída da via vestíbulo espinhal, pois exerce influência tônica ipsilateral nos músculos que atuam sobre os membros inferiores^(10)^. Os casos 1 e 2 não conseguiram realizar a prova, o que corresponde a um comprometimento mais acentuado referente ao equilíbrio dinâmico do que a alteração descrita anteriormente.

Quanto à função cerebelar, os testes evidenciaram presença da dismetria na *Index-Naso* no caso 1 e disdiadococinesia nos casos 1 e 2. Ambas as pacientes apresentaram comprometimento de áreas cerebelares, o que pode justificar tais achados nos testes.

O caso 1 apresentou nistagmo semi-espontâneo horizontal bidirecional, conforme mudança da direção do olhar, enquanto o caso 2 apresentou nistagmo semi-espontâneo horizontal para a esquerda. A presença do nistagmo semi-espontâneo é verificada em lesões cerebelares, compatível com os casos em questão. Esse nistagmo está presente em 20-80% dos casos de EM e ocorre devido defeito na rede integradora neural, estruturas que abrangem o cerebelo, o que provoca incapacidade de manter o olhar em certas posições^(11)^.

Quanto ao nistagmo bidirecional, um estudo^(11)^ registrou a presença em quatro pacientes, sendo horizontal em três casos e oblíquo em um, compatível com o caso 1 deste estudo. A presença desse tipo de nistagmo é um dado relevante para o topodiagnóstico, pois a sua ocorrência em mais de uma posição do olhar é sempre de origem central.

No teste sacádico, todos os casos apresentaram alteração nos parâmetros da velocidade e/ou latência. Um estudo^(11)^ que avaliou 30 indivíduos com EM, constatou anormalidade em todos os indivíduos, em pelo menos um dos parâmetros pesquisados, sendo 96,7% na velocidade, 20% na precisão e 6,7% na latência.

Alterações discretas destes parâmetros podem estar presentes em pacientes com disfunções vestibulares periféricas. Entretanto, no caso 1 foi verificada alteração de morfologia do traçado, a dismetria sacádica do tipo hipermétrica, movimento sacádico que termina aquém do alvo, sugestivo de alterações no cerebelo e no tronco encefálico^(12)^. A paciente deste estudo apresentava lesões em ambas as estruturas.

O VORS, alterado no caso 1, foi sugestivo de hipofunção vestibular à esquerda, compatível com a diminuição da velocidade dos movimentos sacádicos à esquerda. Além disso, o cerebelo é importante para a geração do VORS, principalmente o *vermis* dorsal, o que torna esse teste relevante nas lesões neurológicas^(6)^. O caso 1 apresentou acometimento da região cerebelar, o que está de acordo com a literatura científica.

Foi observado, no teste do impulso cefálico, um predomínio de ganho do RVO reduzido dos CSCs verticais em relação aos laterais nos três casos descritos. Esse padrão não é característico de vestibulopatias periféricas^(13)^, o qual representa, dessa forma, comprometimento das vias vestibulares centrais.

Há três padrões distintos de envolvimento dos CSCs em vestibulopatias unilaterais agudas conforme a inervação: hipofunção dos canais anterior e lateral ou apenas lateral, devido lesão do nervo vestibular superior; hipofunção de todos os canais, devido lesão dos nervos vestibulares superior e inferior; e, hipofunção do canal posterior, devido lesão do nervo vestibular inferior. Assim, quando ocorre dano no labirinto ou em qualquer um dos ramos do nervo vestibular, uma combinação única de déficits é esperada. Dessa forma, hipofunção restrita dos canais verticais (caso 2) ou apenas do anterior (caso 3) não se enquadram como comprometimento exclusivamente periférico, de acordo com esse critério^(13)^.

Ainda que o caso 1 não se adeque a esse padrão supracitado, um estudo^(14)^ descreveu o acometimento isolado de um ou ambos os canais posteriores como resultado de dano no tronco encefálico, compatível com o exame de RMC do caso em questão.

No caso 1, observou-se ganho aumentado no CSC lateral esquerdo. Considerando que não houve calibração deficiente ou excessiva proximidade do alvo e que a paciente passou por reteste e manteve os resultados, o aumento de ganho pode estar relacionado à possível hipersensibilidade do sistema vestibular, que é a percepção anormal quanto à movimentação da cabeça. Essa habilidade geralmente está comprometida em alterações centrais^(15)^.

Referente às sacadas compensatórias, estas foram vistas apenas nos canais verticais, tanto encobertas como evidentes, não só em canais com ganho do RVO reduzido, mas também com ganho dentro dos padrões de normalidade. Um estudo^(2)^ também observou a presença de sacadas em indivíduos com EM.

A elevada incidência de sinais de acometimento vestibular em indivíduos com EM de origem central indica a necessidade de investigação deste sistema na orientação diagnóstica dessa doença. A disfunção vestibular tem, geralmente, origem da junção de comprometimentos diversos, o que torna dificultoso estabelecer as suas causas. O equilíbrio corporal pode ser afetado pelo envolvimento de um ou mais sistemas sensoriomotores que contribuem em sua manutenção, devido as localizações difusas das lesões neurológicas. Dessa forma, é importante que vários testes sejam realizados, em associação, para avaliar o equilíbrio corporal dessa população.

A associação dos achados apresentados sugere a presença de disfunção vestibular central, pois as alterações encontradas nos testes clínicos e oculomotores realizados, como lateropulsão e instabilidade presentes no teste de Romberg e Romberg sensibilizado, alteração no teste sacádico, dismetria, disdiadococinesia, presença do nistagmo semi-espontâneo bidirecional, hipermetria sacádica, alteração do VORS e ganho do RVO deficitário restrito aos CSCs verticais, caracterizam achados compatíveis com afecção central.

Como está evidenciado pelos resultados e estudos aqui discutidos, a disfunção vestibular pode estar presente na EM, visto que as lesões desmielinizantes podem envolver áreas que participam do processamento central das informações vestibulares. Essas áreas integram e interpretam as informações sensoriais que chegam ao SNC^(8)^. Dessa forma, indivíduos com esta doença merecem especial atenção referente à avaliação vestibular e do equilíbrio corporal, pois permitirá direcionar a escolha do melhor tratamento diante dos sinais presentes em cada caso.

O presente estudo evidencia a necessidade de ampliar a pesquisa com maior número amostral para permitir inferências estatísticas. Sugere-se, ainda, que novos estudos possam avaliar indivíduos com diferentes fenótipos da EM, com o objetivo de caracterizar os achados específicos de cada forma clínica e, ainda, empregar outros instrumentos para investigar os diferentes sistemas que controlam o equilíbrio corporal.

## COMENTÁRIOS FINAIS

A associação dos achados obtidos nas provas clínicas, oculomotoras e no teste do impulso cefálico por vídeo, em conjunto com as lesões evidenciadas na RMC em áreas centrais que processam as informações vestibulares, como cerebelo e tronco encefálico, sugerem a presença de disfunção vestibular central nos casos estudados.
